# Targeting Gas6/TAM in cancer cells and tumor microenvironment

**DOI:** 10.1186/s12943-018-0769-1

**Published:** 2018-01-31

**Authors:** Guiling Wu, Zhiqiang Ma, Yicheng Cheng, Wei Hu, Chao Deng, Shuai Jiang, Tian Li, Fulin Chen, Yang Yang

**Affiliations:** 1 Key Laboratory of Resource Biology and Biotechnology in Western China, Ministry of Education. Faculty of Life Sciences, Northwest University, 229 Taibai North Road, Xi’an, 710069 China; 2 Department of Aerospace Medicine, The Fourth Military Medical University, 169 Changle West Road, Xi’an, 710032 China; 3 Department of Thoracic Surgery, Tangdu Hospital, The Fourth Military Medical University, 1 Xinsi Road, Xi’an, 710038 China; 4 Department of Stomatology, Bayi Hospital Affiliated to Nanjing University of Chinese Medicine, Nanjing, Jiangsu 210002 China; 5 Department of Biomedical Engineering, The Fourth Military Medical University, 169 Changle West Road, Xi’an, 710032 China; 6 Department of Cardiovascular Surgery, The First Affiliated Hospital of Xi’an Jiaotong University, 277 Yanta West Road, Xi’an, Shaanxi 710061 China

**Keywords:** Growth arrest-specific 6, Cancer cells, Tumour microenvironment

## Abstract

Growth arrest-specific 6, also known as Gas6, is a human gene encoding the Gas6 protein, which was originally found to be upregulated in growth-arrested fibroblasts. Gas6 is a member of the vitamin K-dependent family of proteins expressed in many human tissues and regulates several biological processes in cells, including proliferation, survival and migration, by binding to its receptors Tyro3, Axl and Mer (TAM). In recent years, the roles of Gas6/TAM signalling in cancer cells and the tumour microenvironment have been studied, and some progress has made in targeted therapy, providing new potential directions for future investigations of cancer treatment. In this review, we introduce the Gas6 and TAM receptors and describe their involvement in different cancers and discuss the roles of Gas6 in cancer cells, the tumour microenvironment and metastasis. Finally, we introduce recent studies on Gas6/TAM targeting in cancer therapy, which will assist in the experimental design of future analyses and increase the potential use of Gas6 as a therapeutic target for cancer.

## Introduction

Gas6 was identified in 1988 and further characterized in mouse embryonic NIH 3 T3 fibroblasts in 1993 [1]. Gas6, a 678-amino acid protein, belongs to the vitamin K-dependent (VKD) family. Gas6 shows the highest affinity for Axl, followed by Tyro3 and then Mer [2]. Gas6 interacts with Tyro3, Axl and Mer (TAM) receptors through its sex hormone-binding globulin (SHBG)-domain and activates downstream signalling, such as phosphatidylinositol 3-kinase (PI3K), extracellular signal-regulated kinase (ERK), and nuclear factor kappa-light-chain-enhancer of activated B cells (NF-κB) pathways, to regulate proliferation, migration, differentiation, adhesion, and apoptosis [3–5].

Numerous studies have shown that upregulation of Gas6/TAM can promote the development of several cancers [6, 7]. Clinically, expression of Gas6 and TAM receptors always predicts a poor prognosis [8]. The results of animal experiments have shown that human prostate cancer cell lines grow significantly better in transplanted vertebral bodies derived from Gas6^−/−^ animals than in those derived from Gas6^+/+^ animals [9]. Gas6/TAM is required for proliferation and migration of cancer cells, and several studies have shown that knockdown of Axl and Mer inhibits tumour cell proliferation and induces apoptosis [10, 11]. Intriguingly, Gas6 also has functions in the tumour microenvironment. Tumours can induce intratumoral macrophages to overexpress and secrete Gas6 by producing interleukin-10 (IL-10) and macrophage colony-stimulating factor (M-CSF) in the microenvironment [12]. In addition, Gas6 binds to TAM receptors on natural killer (NK) cells and inhibits their anti-tumour immune effect [13], and Gas6 can bind to TAM receptors in vascular smooth muscle cells (VSMCs) to inhibit their apoptosis [14]. Interestingly, another study has reported that endogenously produced Gas6 is critical for VSMC proliferation induced by Ca2^+^-mobilizing growth factors but not receptor tyrosine kinases [15]. In this review, we described the Gas6 and TAM receptors and the involvement of Gas6/TAM in different cancers; we then discuss the roles of Gas6 in cancer cells, the tumour microenvironment and metastasis. Finally, we describe recent progress in targeting Gas6/TAM for cancer therapy, which will assist in future experimental design and increase the potential use of Gas6/TAM as a therapeutic target for cancer.

## Gas6/TAM structure and associations with cancer

Gas6 is the product of *growth arrest-specific gene 6* (*gas6*), which was originally identified in fibroblasts as a gene with upregulated expression in growth arrest [16]. Gas6 is characterized by the presence of a C-terminal SHBG-like structure composed of two globular laminin-G-like domains [17]. The N-terminal region contains 11 γ-carboxyglutamic acid residues (Gla), which confer to VKD proteins the ability to bind to anionic phospholipids at the cell surface [3]. Following the Gla domain are a loop region and four epidermal growth factor (EGF)-like domains [3]. TAM receptors (Axl, Mer and Tyro3) are receptor tyrosine kinases with an intracellular tyrosine kinase domain and extracellular domains containing a combination of two N-terminal immunoglobulin (Ig)-like domains and two fibronectin type-III (FNIII) repeats [18–20]. TAM receptors are ectopically expressed or overexpressed in numerous human cancers and are involved in tumour development [21–23]. Significantly, Gas6 regulates proliferation, survival and migration of cancer cells by binding to TAM receptors.

The associations of Gas6 with cancer have been reported in a wide variety of cancers. Specifically, Gas6 is overexpressed in melanoma, schwannoma, glioma and pancreatic ductal adenocarcinoma (PDA) cell lines [6, 10, 24, 25], and several studies have shown that Gas6 is upregulated in ovarian cancer and thyroid cancer specimens from patients [8, 26]. Although there is little available research on the mechanism of Gas6 overexpression, one breast cancer study showed that Gas6 is amplified in breast cancer [27]. Moreover, human prostate cancer cell lines were found to grow significantly better in vertebral bodies transplanted derived from Gas6^−/−^ animals than in those derived from Gas6^+/+^ animals [9, 28]. The functions of TAM receptors in cancers have also been examined in numerous studies. Gas6 binds to Axl and induces cell survival, proliferation and migration [29–33], and overexpression of Axl promotes the development of various types of cancers. Studies have shown that Axl is upregulated in non-small-cell lung cancer (NSCLC), melanoma, osteosarcoma, acute myelocytic leukaemia (AML), schwannoma, glioma, and thyroid cancer cell lines [6, 22, 34–36] and that Axl is overexpressed in tumour tissues from patients with NSCLC, osteosarcoma, AML, and thyroid cancer [21, 22, 34, 35]. To investigate the significance of Axl overexpression in tumour progression, Axl has been knocked down via direct shRNA targeting in several studies. The results showed that knockdown of Axl in PDA and osteosarcoma cells inhibits proliferation and induces apoptosis [10, 11]. Moreover, Axl knockdown in transfected NSCLC and thyroid cancer cells inhibits tumour growth in nude mice [34, 37]. Mer is involved in cancer cell survival and migration. It has been shown that Mer is upregulated in NSCLC, melanoma, AML and schwannoma cell lines [6, 22, 38–40]; Mer is also overexpressed in tumour tissues of patients with NSCLC and AML [22, 41]. Mer overexpression promotes the development of numerous cancers, and knockdown of Mer increases apoptosis in AML cell lines and reduces colony formation in NSCLC and the growth of subcutaneous NSCLC xenografts in nude mice [37, 42]. Several studies have shown that Tyro3 is significantly upregulated in thyroid cancer cells and melanoma cells [24, 34], though few studies have examined the importance of Tyro3 overexpression. Thus, further studies are needed in the future.

## Gas6/TAM in cancer cells

**Fig. 1:**
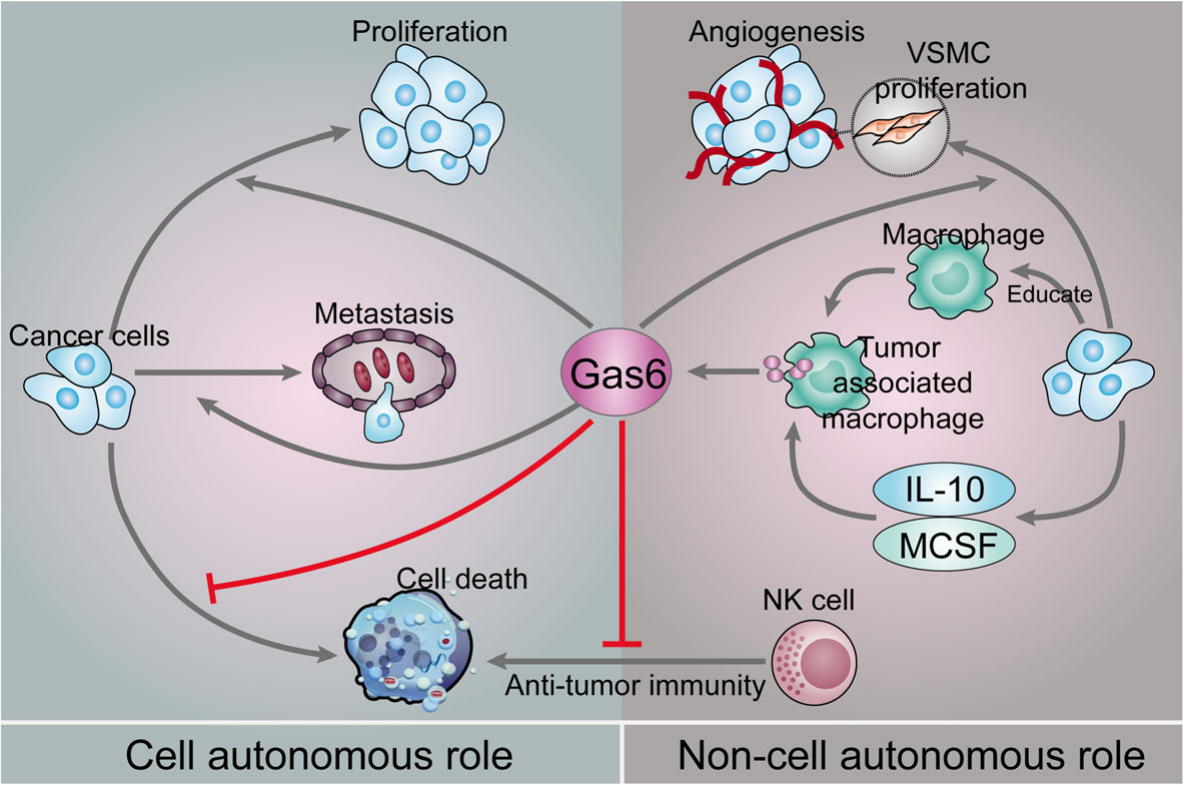
Roles of Gas6/TAM in cancer cells and the tumour microenvironment. Role of Gas6/TAM in cancer cells: Gas6 binds to TAM receptors in cancer cells and promotes their proliferation and migration and inhibits their apoptosis. Role of Gas6/TAM in the tumour microenvironment: Tumours can instruct intratumoral macrophages to overexpress and secrete Gas6 by secreting IL-10 and M-CSF into the microenvironment. Gas6 binds to TAM receptors on NK cells and inhibits their anti-tumour immune effect. Additionally, Gas6 can bind to TAM receptors on VSMCs and promote their proliferation and angiogenesis. Gas6, growth arrest-specific gene 6; IL, interleukin; M-CSF, macrophage colony-stimulating factor; NK, natural killer; VSMC, vascular smooth muscle cell

Within the context of cancer, many studies have described the involvement of Gas6/TAM in the survival, proliferation and migration of a wide variety of cancer cells (Fig. 1). Investigations of the underlying mechanisms indicate the potential of targeting Gas6/TAM as cancer therapy.

### Gas6/TAM in cancer cell survival

Gas6/TAM have a significant role in the survival of cancer cells. For instance, in the osteosarcoma cell lines MG63 and U2OS, Axl activation by recombinant human Gas6 can protect tumour cells from apoptosis caused by serum starvation [21], and knockdown of Axl inhibits osteosarcoma cell proliferation and induces apoptosis. Moreover, when Mer expression in the AML cell lines Nomo-1 and Kasumi-1 was decreased by two independent shRNA constructs, the rate of myeloblast apoptosis increased two- to threefold in response to serum starvation [42]. In this study, researchers also transplanted NOD-SCID-gamma mice with Nomo-1 myeloblasts with reduced levels of Mer, and the results showed significantly prolonged survival of NOD-SCID-gamma mice transplanted with Nomo-1 myeloblasts compared to that of those transplanted with control cell lines. The underlying mechanisms of Gas6/TAM involvement in cancer cell survival should also be studied in the future with regard to genes mutated or upstream regulators. Gas6 and Axl interactions activate the PI3K-protein kinase B (Akt) pathway, which promotes cellular survival [29, 32]. Activation of Akt downregulates Bad, a proapoptotic mediator, and increases the anti-apoptotic protein B-cell lymphoma 2 (Bcl-2) by an NF-κB-dependent mechanism [30]. Gas6 binds to Mer and causes activation of Grb2, which promotes survival through Ras and MEK1 and increased expression of ERK1/2 [43]. Gas6/Mer interaction also activates P38 mitogen-activated protein kinase (MAPK) to promote survival [39], whereas Gas6/Tyro3 stimulates the Akt survival pathway, which leads to nuclear translocation of NF-κB and upregulation of NF-κB target genes (Fig. 1) [6].

### Gas6/TAM in cancer cell proliferation

Gas6 is a mitogen that is upregulated during the G0–G1 phase of the cell cycle [16]. Research concerning the role of Gas6 in different cancers has indicated that Gas6/TAM induces cancer cell proliferation. For instance, Gas6 promotes proliferation of prostate carcinoma cell lines expressing Axl by inducing phosphorylation of Akt and MAPK [28]. Knockdown of Axl reduces the proliferation of osteosarcoma cells, possibly via downregulation of the Akt pathway [21]. In animal experiments, knockdown of Axl in transfected NSCLC and thyroid cancer cells reduces tumour growth in nude mice [34, 37], and Mer knockdown reduces the growth of subcutaneous NSCLC xenografts in nude mice [22]. Another study revealed that the underlying mechanism of schwannoma cell proliferation after Gas6/Axl interaction involves Src, focal adhesion kinase (FAK) and NF-κB [6]. Additional mechanisms involved in the proliferation of cancer cells include the induction of ERK signalling due to Gas6 binding to Axl (Fig. 1) [24].

### Gas6/TAM in cancer cell migration

Migration is induced in several types of cancer, including prostate and breast cancers, in response to Gas6. For instance, Lee et al. studied the DU145 (prostate cancer) and A431 (skin cancer) cell lines and discovered that binding of Gas6 to Axl induces migration mainly by upregulating Slug expression [44]. Expression of Slug, a member of the Snail family, is enhanced in metastatic breast cancer, lung cancer, mesothelioma and melanoma [45] and is involved in metastatic prostate cancer cell invasion and migration [46, 47]. Interestingly, Yang, B et al. found that phosphorylated Axl localizes to active myosin filaments and phosphorylates tropomyosin at a tyrosine residue critical for adhesion formation, which may indicate the involvement of Axl in neoplastic migration [48]. However, this process is ligand-independent (Fig. 1).

## Gas6/TAM in the tumour microenvironment

Above, we describe cell autonomous roles of Gas6/TAM in cancer cells. In this section, the non-cell-autonomous roles of Gas6/TAM in the tumour microenvironment, including the involvement of immune cells and VSMCs, will be summarized. The tumour microenvironment is the cellular environment in which the tumour exists, including surrounding blood vessels, immune cells, fibroblasts, bone marrow-derived inflammatory cells, lymphocytes, signalling molecules and the extracellular matrix. The tumour and the surrounding microenvironment are closely related and constantly interact with each other. Tumours can influence the microenvironment by releasing extracellular signals, promoting tumour angiogenesis and inducing peripheral immune tolerance, and B cells in the microenvironment can affect the growth and evolution of cancerous cells, such as in immune editing [49]. The Gas6/TAM axis affects the tumour microenvironment by modulating diverse cellular functions, including those of immune cells and VSMCs [50]. Below, we discuss the roles of Gas6/TAM in immune cells and VSMCs in the tumour microenvironment (Fig. 1).

Several publications have demonstrated the roles of Gas6/TAM in immune cells. For instance, tumours can instruct intratumoral macrophages to overexpress and secrete Gas6 by producing IL-10 and M-CSF in the microenvironment [12], and Gas6 binds to TAM receptors in tumour cells and immune cells, promoting tumour progression. Additionally, a recent study demonstrated a role for TAM in anti-tumour immunity involving NK cells [13]. Due to the diversity of macrophage functions, several attempts have been initiated to categorize these cells, resulting in one commonly used classification based on their immunogenic function [51, 52]: macrophages that enhance inflammation are known as M1 macrophages, whereas those that decrease inflammation and enhance tissue repair are known as M2 macrophages [53]. Intriguingly, tumour-associated macrophages are derived from circulating monocytes or resident tissue macrophages, which constitute the major leukocytic infiltrate in the tumour microenvironment [54], and tumour-associated macrophages cannot be classified as M1 or M2 macrophages because they exhibit lower levels of expression of M1- and M2-related factors [55]. Tumour-associated macrophages interact with a wide range of growth factors, cytokines and chemokines in the tumour microenvironment, which is believed to instruct the macrophages and determine their specific phenotype; hence, their functional roles may differ because the microenvironment varies between different types of tumours [56]. One study demonstrated the role of macrophage-derived Gas6 in experimental models of solid tumours, including colorectal cancer and breast cancer [12]. In this study, tumour cells did not express Gas6, whereas CD45 infiltrating-infiltrating leukocytes showed abundant expression of this protein. In addition, the Gas6 expression level in these leukocytes was found to be specifically upregulated after entering the tumour, as these cells do not secrete Gas6 while circulating in the blood or residing in the bone marrow. Further analysis revealed that tumour-associated macrophages are the primary source of Gas6 within the tumour microenvironment. In contrast, tissue-resident macrophages isolated from lungs or from the peritoneum expressed much lower levels of Gas6 than tumour-associated macrophages. Thus, crosstalk between tumours and macrophages leads to specific upregulation of Gas6, which promotes tumour progression [12]. Moreover, as mentioned above, macrophages in the tumour microenvironment can be instructed and polarized, and Chiu et al. discovered that Axl signalling is involved in the polarization of tumour-associated macrophages towards an M2 phenotype, with increased M2 surface marker and gene expression, in oral squamous cell carcinoma [7]. The underlying mechanism may involve the Axl/PI3K/Akt/NF-κB pathway. Intriguingly, an in vitro study showed that NK cells express each of the TAM receptors and that NK cell proliferation and IFN-γ production are both inhibited after Gas6 binds to TAM receptors [13]. The results reported by these authors from experiments using LDC1267, a highly selective TAM inhibitor, indicated that TAM receptor inhibition in vivo releases NK cells to kill tumour cells. Moreover, in vivo therapy with LDC1267 in different model systems and via different routes of administration consistently reduces metastasis, and this effect is dependent on NK cells. All of these results may provide a rationale for future research targeting TAM and promoting anti-tumour immunity for the treatment of cancer.

VSMCs are a component of the tumour microenvironment, and proliferation of VSMCs promotes tumour angiogenesis [57]. TAM receptors and ligands are expressed in vascular smooth muscle cells, and the Gas6–Axl pathway has been implicated in vasculogenesis [58]. For example, Axl^−/−^ mice exhibit defects in vessel permeability and integrity, but Mer^−/−^ and Tyro3^−/−^ mice do not display such defects, which again emphasizes the different roles of TAM receptors, with Axl promoting angiogenesis in the vasculature [59]. Several studies have shown that Gas6 and Axl are involved in angiogenesis. Melaragno et al. observed that treatment of VSMCs with 100 ng/ml Gas6 decreased VSMC apoptosis [14]. The ability to prevent apoptosis requires both Gas6 binding to Axl and Axl kinase activity, as treatment with a soluble, competitive Axl extracellular domain protein or transfection of a kinase inactive mutant (Axl-K567R) was found to completely prevent the anti-apoptotic effect.

## Gas6 in cancer metastasis

Metastasis, or metastatic disease, is the spread of cancer from one organ to another and is a typical feature of malignant cells [60]. Cancer cells can circulate through the bloodstream or lymphatic system to other tissues in the body. Gas6 promotes metastasis by regulating invasion [44]. The invasive nature of tumour cells is the major prerequisite for cancer metastasis, and Gas6 is clearly involved in cancer invasion. Using DU145 (prostate cancer) and A431 (skin cancer) cells, Lee et al. demonstrated that Gas6 reduces E-cadherin expression and induces expression of vimentin [44]. An immunoblot analysis conducted in this study revealed that E-cadherin expression was substantially reduced in DU145 cells in a dose-dependent manner following treatment with conditioned medium containing a recombinant human Gas6 fusion protein or purified recombinant Gas6 protein compared to that following treatment with mock-conditioned medium or no treatment, respectively. Downregulation of E-cadherin and upregulation of vimentin are well-known characteristics of epithelial–mesenchymal transition (EMT) [61], during which epithelial cells gradually lose their epithelial structures, such as E-cadherin-mediated cell-cell adhesion, while concomitantly acquiring mesenchymal characteristics, such as upregulated expression of vimentin [62]. The mechanism underlying this process has been clarified. By phosphorylating activator protein-1 and the transcription factor c-Jun as well as activating transcription factor-2, Gas6/Axl prompts c-Jun N-terminal kinase (JNK) and ERK1/2 signalling in cancer cells; this results in the induction of Slug, which is an E-cadherin transcriptional repressor that belongs to the Snail superfamily of zinc-finger factors and is required for cell migration [44, 63]. Another study also indicated that the Gas6/Axl axis induces the invasion of prostate cancer cells to the bone marrow and enhances cell survival during metastasis [64]. In breast cancer, Axl is required for cell invasion as well as EMT and cancer progression, and it drives cell migration, neovascularisation, and tumour growth [65, 66]. Additionally, malignant cells fuel tumour growth by stimulating infiltrating leukocytes to produce the mitogen Gas6 [12]. Interestingly, Gas6 promotes prostate cancer cell dissemination to the bone marrow [64]. Once these cells have migrated to the bone marrow, disseminated tumour cells (DTCs) may lie dormant for years, undetected by standard clinical methods. The quiescence of these DTCs in the bone marrow may be regulated by micro-environmental controls derived from cellular ‘niches’ predominately comprising osteoblasts, endothelial cells and other marrow elements. Prostate cancer tissues and osteoblasts in “niches” can secrete Gas6, which binds to TAM receptors expressed by prostate cancer cells and induces prostate cancer dormancy. Additionally, Dormady et al. found that Gas6 plays an important role in supporting pluripotential hematopoietic stem cells in bone marrow. The role of Gas6 in hemopoietic system indicated by this article may promote us to understand the effect of Gas6 in tumors of hematopoietic and lymphoid tissues or hematopoietic and lymphoid malignancies [67]. In summary, Gas6 promotes metastasis by regulating invasion.

## Research progress in targeting Gas6/TAM for cancer therapy

Upregulation of Gas6/TAM and their role in promoting cancer cell survival, proliferation and migration in numerous types of cancers suggests potential Gas6/TAM therapeutic targets. Several studies have shown that knockdown of Axl in PDA and osteosarcoma cells inhibits tumour cell proliferation and induces apoptosis [10, 11]. Additionally, Axl knockdown in transfected NSCLC and thyroid cancer cells inhibits tumour growth in nude mice [34, 42]; knockdown of Mer increases apoptosis in AML cell lines and reduces colony formation in NSCLC as well as the growth of subcutaneous NSCLC xenografts in nude mice [22, 40]. Intriguingly, the immunosuppressive effects of Gas6/TAM in tumour microenvironments also provide effective cancer treatment through the inhibition of Gas6/TAM. The innate immune system has TAM receptor-mediated safeguards to prevent prolonged and injurious inflammation, although few studies have directly explored the role of TAM signalling in the context of tumour immunology. Nevertheless, as noted by Paolino in their review [67], these limited studies have indicated a central role for TAM receptors and their ligands in the regulation of anti-tumour immunity [12, 13, 68]. One study found that the absence of Mer receptors markedly increased serological levels of inflammatory cytokines and led to higher levels of immune cells in the tumour microenvironment [68]. Intratumoural CD8^+^ T lymphocyte numbers were higher in tumour-bearing Mer^−/−^ mice than in tumour-bearing wild-type mice, and antibody-mediated CD8^+^ T lymphocyte depletion restored tumour growth in Mer^−/−^ mice. These results indicate that targeting Mer in the tumour microenvironment may have clinical benefits, enhancing anti-tumour immune responses and promoting immunotherapeutic strategies [68]. Recently, Axl and Mer inhibitors have been further investigated, and several small selective inhibitors have advanced into clinical trials. A “decoy receptor” was designed to inhibit Gas6/Axl signalling, which may have the potential to inhibit tumour development in vivo [69]. Below, we discuss numerous results associated with Gas6- and TAM-targeting treatments (Table 1).

**Table 1:** Research on targeting Gas6/TAM for cancer therapy

	Therapy	Target	Cancer type	Reference
Specific	R428	Axl	Breast cancer, GBM, AML and Ewing sarcoma	[74, 76–78]
	UNC1062	Mer	melanoma	[39]
	UNC2025	Mer	GBM	[87]
	Mer590	Mer	NSCLC	[88]
Non-specific	shRNA	Axl	Breast carcinoma	[65]
	shRNA	Mer	Melanoma and AML	[39, 42]
	siRNA	Tyro3	Breast cancer	[23]

*Gas6* growth arrest-specific gene 6, *AML* acute myeloid leukaemia, *GBM* glioblastoma multiforme, *NSCLC* non-small-cell lung cancer

### Therapy targeting Gas6

As noted above, Gas6 plays a significant role in the development of numerous cancer types [8, 68, 69]. Moreover, Gas6 is involved in resistance to cancer therapy [70, 71]. In a study in which plasma DNA was sequenced to analyse acquired resistance to cancer therapy, Gas6 was found to contribute to resistance to breast cancer therapy [70]. An increase in the abundance of a splicing isoform of Gas6 was observed after further treatment of one patient with lapatinib in combination with capecitabine [72]. In the same study, activation of the Axl kinase pathway was found to cause resistance to tyrosine kinase inhibitors in NSCLC as well as resistance to lapatinib in estrogen receptor-positive (ER-positive), human epidermal growth factor receptor-2 (HER 2)-positive breast cancer cell lines. All of these findings suggest that targeting Gas6 may be an effective approach to treating tumours. Therefore, targeting Gas6 may effectively aid existing cancer treatments. Although there is no existing research on Gas6-specific inhibitors, a recent study exploited a novel way of inhibiting Gas6/Axl signalling, which may inspire the development of better cancer therapies [73]. The authors engineered an Axl ‘decoy receptor’ that binds to Gas6 with high affinity to inhibit its function, offering an alternative approach to drug discovery efforts that directly target Gas6. Four mutations within the high-affinity Axl variant cause structural alterations in side chains across the Gas6-Axl binding interface, stabilizing a conformational change in Gas6. When reformatted as an Fc fusion, the engineered decoy receptor binds to Gas6 with femtomolar affinity, constituting an 80-fold improvement compared with the binding of the wild-type Axl receptor and allowing effective sequestration of Gas6 and specific abrogation of Axl signalling. Moreover, this increased Gas6 binding affinity is critical and correlates with the ability of decoy receptors to potently inhibit metastasis and disease progression in vivo. The results suggest a novel method for inhibiting Gas6/Axl signalling [73].

### Therapy targeting the Axl receptor

Accumulating evidence suggests important roles for the Axl receptor tyrosine kinase in cancer progression, invasion, metastasis, drug resistance, and patient mortality, highlighting Axl as an attractive target for therapeutic development [21, 25]. For example, Axl is highly expressed in invasive breast cancer cells, and Axl knockdown blocks the invasive phenotype. Moreover, high Axl expression in primary breast tumours is a strong independent predictor of poor patient outcomes [74]. As mentioned in Graham et al.’s review, a wide range of small-molecule kinase inhibitors that target the Axl receptor have been described in several studies, including Foretinib, Cabozantinib, Merestinib, Bosutinib, Gilteritinib, Crizotinib, Amuvatinib, Sunitinib, MGCD265, ASLAN002, NPS-1034, LDC1267, SGI-7079, TP-0903, UNC2025, S49076 and BGB324 [75]. However, in most cases, Axl was not the intended primary target but a secondary target resulting from the similarities among the kinase domains of Axl and other receptor tyrosine kinases (RTKs), such as MET or Mer. Consequently, these inhibitors often show less potency for Axl than their main target. Intriguingly, BGB324, also known as R428, was found to be an Axl-selective inhibitor, and has advanced to clinical trials [74]. R428 inhibits Axl with low nanomolar activity and blocks Axl-dependent events, including Akt phosphorylation, breast cancer cell invasion, and proinflammatory cytokine production. Pharmacologic investigations have revealed favourable effects after oral administration, with R428-treated tumours displaying a dose-dependent reduction in expression of the cytokine granulocyte macrophage colony-stimulating factor and the epithelial-mesenchymal transition transcriptional regulator Snail. In agreement with an earlier study, R428 inhibited angiogenesis in corneal micropocket and tumour models. Furthermore, R428 administration reduced the metastatic burden and extended survival in MDA-MB-231 intracardiac and 4 T1 orthotopic mouse models of breast cancer metastasis. Additionally, R428 acted synergistically with cisplatin to enhance suppression of liver micrometastases [74]. Notably, in addition to breast cancer, R428 has been shown to inhibit Axl signalling in glioblastoma multiforme (GBM), AML and Ewing sarcoma, indicating the effectiveness of R428 for targeting Axl [76–78]. Indeed, R428 is now in clinical development [77], and several ongoing controlled trials involving R428 at various clinical centres aimed at identifying its maximum tolerated dose are registered at ClinicalTrails.gov (Identifier: NCT02922777, NCT02488408, NCT02424617 and NCT02872259). These studies include trials of R428 in NSCLC, AML and metastatic melanoma, and results are expected soon. In addition to specific inhibitors, shRNA knockdown of Axl and inhibition of Axl with siRNA are also effective approaches to inhibiting Axl signalling [22, 79]. In fact, the first clinical trial using R428 for the treatment of acute myeloid leukaemia and non-small cell lung cancer is currently being conducted [80], and several other new inhibitors specific for TAM receptors are being tested in a preclinical stage [81, 82]. Stable shRNA knockdown of Axl significantly reduces tumour growth in a xenograft model of breast carcinoma [65], and inhibition of Axl with siRNA in human umbilical vein endothelial cells blocks endothelial tube formation in vitro, suggesting that inhibition of Axl may restrict the angiogenesis required for breast tumour cell growth [79]. Several monoclonal antibodies specifically targeting AXL have been reported, including 12A11 [83], Mab173 [84], YW327.6S2 [85] and, more recently, D9 and E8 [86]. All these results may provide avenues for potential therapeutic targeting of Axl.

### Therapy targeting Mer

Mer is often overexpressed or activated in various malignancies with oncogenic properties, and several studies on Mer-targeted therapies have been performed. For instance, treatment of melanoma cells with UNC1062, a novel Mer-selective small-molecule tyrosine kinase inhibitor, has been shown to reduce Mer-mediated downstream signalling activation, induce apoptosis in culture, reduce colony formation in soft agar, and inhibit the invasion of melanoma cells [39]. Moreover, an improved Mer-selective small-molecule tyrosine kinase inhibitor, UNC2025, has been shown to exert anti-tumour effects in GBM lines [87]. These findings establish Mer as a therapeutic target in melanoma and provide a rationale for the continued development of Mer-targeted therapies. shRNA targeting of Mer is used to reduce Mer-mediated downstream signalling, which decreases colony formation by up to 59% and diminishes tumour volume by 60% in a human melanoma murine xenograft model [39]. Furthermore, shRNA constructs have been used to decrease Mer expression in the AML cell lines Nomo-1 and Kasumi-1, and this reduction in Mer protein significantly increases the rate of myeloblast apoptosis by two- to threefold in response to serum starvation [42]. Mer590, a novel monoclonal antibody targeting Mer, has also shown effective results, rapidly and robustly reducing surface and total Mer levels in multiple cell lines. Mechanistically, Mer downregulation is mediated by receptor internalization and degradation, leading to inhibition of downstream signalling through Akt and ERK1/2. Functionally, these effects result in increased apoptosis, increased chemosensitivity to carboplatin, and decreased colony formation. In addition to carboplatin, Mer590 interacts cooperatively with shRNA-mediated Mer inhibition to augment apoptosis [88].

### Therapy targeting Tyro3

Studies in which Tyro3 is targeted are rare. However, Ekyalongo et al. did find that Tyro3 knockdown using siRNA induced the greatest suppression of proliferation in ER-positive/HER2-non-amplified (luminal-type) cells and, to a lesser extent, in ER-negative/HER2-amplified (HER2-type) cells [23]. Conversely, no inhibition of proliferation was observed in ER-negative/HER2-non-amplified (triple-negative type) cells [23]. Such proliferation suppression is correlated with G0-G1/S-phase arrest. These findings indicate the effectiveness of targeting Tyro3 in breast cancer and the feasibility of Tyro3 knockdown via siRNA.

## Conclusions

In our review, we have described the significant roles of TAM receptors in cancer development, including in cancer progression, local invasion and metastasis. Many studies have shown the ligand-dependent activity of TAM receptors in cancer development; however, few studies have focused on ligand-independent activity of TAM. An interesting finding regarding ligand-independent activity of Axl is that this ligand-independent activity diversifies EGFR-induced signalling into additional downstream pathways beyond those triggered by EGFR alone, and this additional signalling may underlie EGFR inhibitor resistance [86]. The proteins S1 and Gas6 are two vitamin K-dependent ligands, and several additional TAM ligands, including TUBBY, TUBBY-like protein 1 (TULP-1) and Galectin-3, have recently been reported [87, 88]. The association of Gas6 with cancer has been summarized thoroughly in this review. Though the protein S1 was overexpressed in AML [89], thyroid cancer [90], ovarian cancer [91], pancreatic cancer [92], brain tumours [93], lung cancer [94], prostate cancer [95], colorectal cancer [96] and osteosarcoma [97]. The S1 protein has only been shown to be involved in prostate cancer metastasis and is associated with prostate cancer prognosis, but the involvement of TAM receptors in these processes has not been shown [95]. Studies concerning the effects of TUBBY and TULP-1 in cancer are rare. Decreased galectin 3 expression relative to that of normal cells or adjacent tissues has also been reported in multiple tumour types [98, 99]. Similarly, increased galectin 3 expression has been associated with a more favourable prognosis in several tumour types [100, 101]. However, the cross reactivity between the ligands and ligand-receptors has barely been investigated; therefore, further studies concerning the cross reactivity among these ligands will provide new insights into how to systematically target Gas6/TAM signalling.

Upregulation of Gas6/TAM and their role in promoting cancer cell survival, proliferation and migration in numerous types of cancers suggest Gas6/TAM therapeutic targets. Moreover, immunosuppressive effects of Gas6/TAM in tumour microenvironments also make inhibition of Gas6/TAM effective in treating cancer. Knockdown of Gas6 and TAM receptors by shRNA, selective small-molecule inhibitors and non-specific inhibitors is being investigated, and several ongoing trials involving R428 at various clinical centres are registered at ClinicalTrails.gov [38, 69, 75]. These results may aid in future experimental design and increase the potential use of Gas6/TAM as therapeutic targets for cancer. Intriguingly, TAM receptors activated by their ligands regulate numerous cellular functions throughout physiological development and adulthood [102]. Expressed by a wide range of cell types and tissues, they play significant roles in homeostatic regulation of the immune, nervous, vascular, bone and reproductive systems [102]. The loss-of-function of TAM signalling in adult tissues is involved in the destruction of tissue homeostasis and diseased states, while TAM gain-of-function in various tumours promotes cancer phenotypes [67]. Therefore, prolonged inhibition of TAM receptors remains controversial. Axl and Mer double-knockout mice displayed enhanced colitis [103], and Bosurgi et al. demonstrated that although Axl and Mer can function as oncogenes in a number of cancers; these genes play a protective role against the development of colitis-associated cancer [104]. These findings underscore the potential adverse effects of systemic inhibition of Axl and Mer and highlight the importance of developing therapeutic strategies to spare these RTKs in macrophage populations relevant to the regulation of local inflammation and tissue homeostasis. Further studies focusing on the local inhibition of TAM receptors may be helpful in identifying TAM inhibitors with fewer side effects. In this framework, and based on the overall literature herein presented, we would like to reinforce the potential therapeutic use of Gas6/TAM signaling inhibition for cancer therapy. We anxiously await future research that could help translate the exciting experimental observations into the clinics.

## Abbreviations

Akt: Protein kinase B
AML: Acute myelocytic leukaemia
Bcl-2: B-cell lymphoma 2
DTCs: Disseminated tumour cells
EGF: Epidermal growth factor
EMT: Epithelial–mesenchymal transition
ER: Estrogen receptor
ERK: Extracellular signal-regulated kinase
FAK: Focal adhesion kinase
FNIII: Fibronectin type-III
Gas6: Growth arrest-specific 6
GBM: Glioblastoma multiforme
HER 2: Human epidermal growth factor receptor-2
Ig: Immunoglobulin
IL-10: Interleukin-10
JNK: C-Jun N-terminal kinase
MAPK: Mitogen-activated protein kinase
M-CSF: Macrophage colony-stimulating factor
NF-κB: Nuclear factor kappa-light-chain-enhancer of activated B cells
NK: Natural killer
NSCLC: Non-small-cell lung cancer
PDA: Pancreatic ductal adenocarcinoma
PI3K: Phosphatidylinositol 3-kinase
RTKs: Receptor tyrosine kinases
SHBG: Sex hormone-binding globulin
TAM: Tyro3, Axl and Mer
TULP-1: Tubby-like protein 1
VKD: Vitamin K-dependent
VSMCs: Vascular smooth muscle cells
